# Supplementary material for: Effect of Ketogenic Diets on Body Composition and Metabolic Parameters of Cancer Patients: A Systematic Review and Meta-Analysis
Source: Nutrients. 2022 Oct 8;14(19):4192. doi: 10.3390/nu14194192 (PMC9570668; doi:10.3390/nu14194192)
Supplement: Supplementary file 1 [file nutrients-14-04192-s001.zip › Suplementary Table S2 Characteristics of included studies.pdf]

Author, year	Design	Inclusion criteria	Exclusion criteria	Intervention duration	Control diet	Intervention	Treatment	Outcome
Cohen 2018	RCT	Women with ovarian or endometrial cancer, BMI≥18.5 kg/m2, age ≥19 years, no medical condition affecting body weightt (other than cancer and associated treatment), and not attempting diet modification.	Not mentioned	12 weeks	American Cancer Society diet: promoting fiber-rich fruits, vegetables, whole grains, and lean meats with small amounts of healthful fats.	Ketogenic diet, approximately 5% of energy from carbohydrate (≤20 g/day), 25% from protein (≤100 g/day) and 70% from fat (≥125 g/day).	Chemotherapy	Short Form-12 Health Survey, dietary adherence, body composition, metabolic effects, serum lipids, serum ketone levels, adverse events
OK 2018	RCT	Adults diagnosed with ancreatobiliary cancer and hospitalized to undergo pancreaticoduodenectomy or distal pancreatectomy	Pregnant, severe diabetes patients with diabetic complications, hyperlipidemia patients with cardiovascular complications, patients with renal impairment below 90% of normal glomerular filtration rate, and illiterate patients	10 days	General Diet: carbohydrate: protein: fat ratio of 55-65: 7-20: 15-30	Ketogenic diet: 3-6% of daily energy as carbohydrates;1g/kg of high-quality protein; 70-80% of energy through fats; Fat: Carbohydrate+Protei n, (w:w) =1.05-1.75:1.	Pancreatectomy	Subject characteristics, patient-generated subjective global assessment, biochemical indices, meal satisfaction score and meal intake-related problems, body composition, urine ketones

Kang 2019	RCT	Adult patients (≥ 19 years old) with pancreatobiliary cancer who underwent pancreaticoduodenectomy or distal pancreatectomy	Pregnancy. , illiterate, foreigners, severe diabetic complications, hyperlipidaemia with cardiovascular complications, or renal insufficiency with a normal glomerular filtration rate<90%.	4 weeks	General diet: 55–65%, 7–20%, and 15–30% of energy from carbohydrate, protein, and fat.	Low-carbohydrate ketogenic diet: 4%, 16%, and 80% of energy from carbohydrate, protein, and fat.	Pylorus-preserving pancreatico-duodenectomy, distal pancreatectomy	Blood biochemistry, ketone bodies, insulin, glucose, and TNF-α in blood, differential metabolites
Khodabakhshi 2019	RCT	Breast cancer patients with locally advanced or metastatic disease, between 18 and 70 years old, and undergoing chemotherapy for at least 3 months.	Patients with significant cardiac, renal or neurologic comorbidities, or an active state of malnutrition, diabetes, pregnancy, and Karnofsky index less than 70.	3 months	Standard diet:55% CHO, 15% protein, and 30% fat.	A medium chain triglycerides (MCT)-based KD (contained 6% calorie from CHO, 19% protein, 20% MCT, 55% fat)	Deprivation therapy	Safety and tolerability of diet, quatity of life, dietary intake and adherence, survival rate, body composition and biochemical parameters

Klement 2019	Non-RCT	Head and neck cancer patients. Age between 18 and 75 years. 18 kg/m2<BMI<34 kg/m2. Karnofsky index ≥70.	Palliative patients, in particular known metastases; type I diabetes; pregnancy; pacemaker and other metallic parts that influence BIA measurements. Known defects in enzymes necessary for ketogenesis, ketolysis, fatty acid oxidation or gluconeogenesis. Unable to speak or understand German. Cognitive impairments or psychological disorders. Renal insufficiency.	5-6 weeks	Standard diet	Ketogenic diet with essential amino acids supplement.	Chemotherapy	Change of body composition
Augustus 2020	RCT	Diagnosed with cancer>1 year, currently receiving chemotherapy or radiation treatment, being>18 years of age, currently a nonvegetar-ian and currently consuming a carbohydrate-based diet (>40%)	Subjects currently on a ketogenic treatment plan, disease, or medical conditions which may affect usual dietary behavior (diabetes, renal disease, stomach ulcers, and hypertension) and preexisting psychosocial conditions such as moderate/severe depression/anxiety.	16 weeks	Standard traditional diet	Modified ketogenic diet contained approximately 10% CHO, 15% proteins, and 75% fats.	Chemotherapy or radiation treatment	Quality of life, biochemical measures, body composition.

Klement 2020	Non-RCT	Women between 18 and 75 years of age with non-metastatic breast cancer	Karnofsky index < 70, body mass index (BMI) < 18 kg/m2, metallic implants, pregnancy, cognitive impairment, inability to speak or understand German, and metabolic defects posing a contraindication against consuming a KD.	18–47 days	Standard diet: whole grains, vegetables, and fruits and limiting fats to 30–35% daily energy intake, with an emphasis on reducing fats from animal origin.	Ketogenic diet with essential amino acids supplement, suggesting replacing carbohydrates with fat, consuming 75–80% calories from fat, and limiting carbohydrates to 50 g per day and 10 g per meal.	Radiotherapy	Dietary adherence, body composition, safety and quality of life, blood parameters.
Voss,2020	RCT	Recurrence of a histologically confirmed glioblastoma, gliosarcoma or malignant progression, of a lower grade glioma on MRI. Age above 18 years and Karnofsky performance score (KPS) ≥60%. Prior radiation therapy of the tumor at least 6 months before inclusion, prior therapy with temozolomide, multidisciplinary tumor board recommendation for re-irradiation. Adequate hematologic, hepatic, renal and coagulatory function.	Bowel obstruction. Malnutrition, cachexia, insulin-dependent diabetes or other medical conditions that might increase the risk of the dietary intervention or could impair the ability to adhere to the diet.	9 days	Standard diet without calorie restriction	Two calorically restricted ketogenic diet 3-day intervals flanking 3 days of fasting. Calorie restriction was defined as 21-23 kcal per kg body weight, Carbohydrates were limited to 50 g/d.	Radiation therapy	Body weight and quality of life, metabolic parameters, PFS/OS, adverse effects.

Kämmerer2021	Non-RCT	Women aged between 26–69 years (mean age 51.7 years) were enrolled in the study during standard rehabilitation at one specific center after the treatment of primary or recurrent BC.	Karnofsky index < 70 and/or expected life span 12 months, additional malignant tumors at the time of recruitment, participation in other trials, unintentional weight loss and body mass index (BMI) < 18, dementia or other clinically relevant alterations of mental status that could impair the ability to cope with the diet, insufficient knowledge of the German language and therefore inability to follow instructions, type 1 diabetes mellitus, decompensated heart failure, myocardial infarction within the last 6 months, symptomatic atrial fibrillation, severe acute infection, pregnancy or pancreatic insufficiency.	20 weeks	Standard diet: CHO 52-62%, Protein 16-17%, Fat 28-31%	KD: CHO 2-4%, Protein 16-18%, Fat 80-85%	Multimodal interventions	Physical performance, body composition, quality of life, blood parameters.

Klement 2021	Non-RCT	Age between 18 and 75 years, diagnosis of a non-metastatic colorectal cancer, Karnofsky index ≥70 and body mass index (BMI) between 18 and 34 kg/m2.	Metallic implants, pregnancy, cognitive impairment, inability to speak or understand German and metabolic disorders that would pose a counter-indication for a KD.	30-55 days	Standard diet	Ketogenic diet (≤50 g of carbohydrates per day) with essential amino acids supplement.	Radiation therapy，chemotherapy	Body composition, pathological tumor responses, blood parameters, quatity of life.
