# Supplementary material for: Effect of Ketogenic Diets on Body Composition and Metabolic Parameters of Cancer Patients: A Systematic Review and Meta-Analysis
Source: Nutrients. 2022 Oct 8;14(19):4192. doi: 10.3390/nu14194192 (PMC9570668; doi:10.3390/nu14194192)
Supplement: Supplementary file 1 [file nutrients-14-04192-s001.zip › Supplementary Figures S1-5.pdf]

Figure S1 Supplementary analysis of effects on body composition

(A) Body weight: post-intervention versus baseline

Study or Subgroup	Ketogenic diet Mean	SD	Total	Non-ketogenic diet Mean	SD	Total	Weight	Std. Mean Difference IV, Random, 95% CI
**1.1.1 Baseline**								
Augustus 2020	64.36	9.95	16	62.87	8.93	20	14.4%	0.16 [-0.50, 0.81]
Cohen 2018	81.2	4.5	25	89	7.1	20	0.0%	-1.32 [-1.98, -0.67]
Kämmerer 2021	67.2	11.83	20	69.06	14.03	25	18.0%	-0.14 [-0.73, 0.45]
Kang 2019	63.82	29.95	9	56.53	23.51	9	7.2%	0.26 [-0.67, 1.19]
Khodabakhshi 2019	71.7	11.9	30	70.5	17	30	24.3%	0.08 [-0.43, 0.59]
Klement 2019	67.23	9.819	5	73.59	14.62	17	6.1%	-0.44 [-1.45, 0.56]
Klement 2020	76.22	15.7	29	71.32	17.39	30	23.7%	0.29 [-0.22, 0.80]
OK 2018	64.6	11.2	9	56.2	7.2	8	6.2%	0.84 [-0.17, 1.84]
**Subtotal (95% CI)**			**118**			**139**	**100.0%**	**0.13 [-0.12, 0.38]**
Heterogeneity: Tau² = 0.00; Chi² = 4.44, df = 6 (P = 0.62); I² = 0%								
Test for overall effect: Z = 1.01 (P = 0.31)								
**1.1.2 Post intervention**								
Augustus 2020	60.49	9.16	16	63.21	9.07	20	14.3%	-0.29 [-0.95, 0.37]
Cohen 2018	75.1	4.5	25	86	7.2	20	0.0%	-1.83 [-2.54, -1.12]
Kämmerer 2021	64.34	10.02	20	66.41	12.96	25	18.0%	-0.17 [-0.76, 0.42]
Kang 2019	60.97	26.71	9	53.68	22.43	9	7.2%	0.28 [-0.65, 1.21]
Khodabakhshi 2019	65.4	11.1	30	69.2	17.3	30	24.2%	-0.26 [-0.77, 0.25]
Klement 2019	62.8	12.37	5	68.93	13.72	17	6.2%	-0.44 [-1.45, 0.57]
Klement 2020	73.81	14.76	29	72.2	17.8	30	24.0%	0.10 [-0.41, 0.61]
OK 2018	60.6	9.5	9	52.7	7.2	8	6.1%	0.88 [-0.13, 1.89]
**Subtotal (95% CI)**			**118**			**139**	**100.0%**	**-0.06 [-0.31, 0.19]**
Heterogeneity: Tau² = 0.00; Chi² = 5.95, df = 6 (P = 0.43); I² = 0%								
Test for overall effect: Z = 0.51 (P = 0.61)								

Test for subgroup differences: Chi² = 1.15, df = 1 (P = 0.28), I² = 13.3%

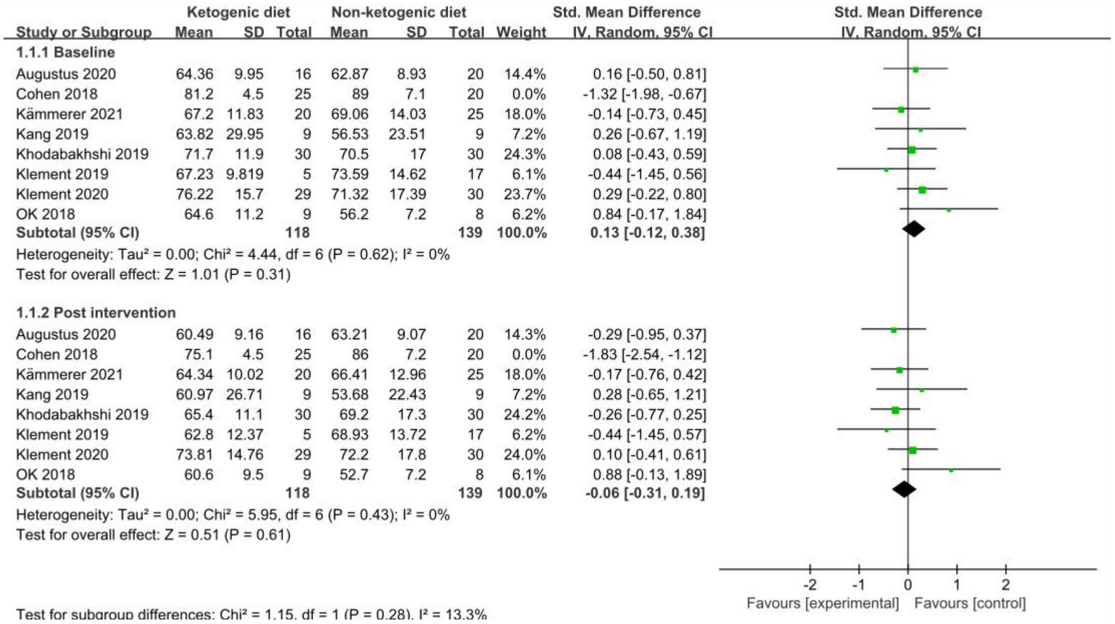

(B) Body weight: change from baseline

Study or Subgroup	Ketogenic diet Mean	SD	Total	Non-ketogenic diet Mean	SD	Total	Weight	Std. Mean Difference IV, Fixed, 95% CI
Klement 2019	-4.43	2.95	5	-4.66	4.71	17	0.0%	0.05 [-0.95, 1.05]
Klement 2020	-2.86	2.02	29	0.46	1.27	30	57.1%	-1.95 [-2.58, -1.32]
Klement 2021	-4.02	2.83	18	-0.08	1.84	23	42.9%	-1.66 [-2.39, -0.94]
Voss 2020	-2.1	1.8	20	-0.7	1.4	22	0.0%	-0.86 [-1.49, -0.22]
**Total (95% CI)**			**47**			**53**	**100.0%**	**-1.83 [-2.30, -1.35]**
Heterogeneity: Chi² = 0.34, df = 1 (P = 0.56); I² = 0%								
Test for overall effect: Z = 7.55 (P < 0.00001)								

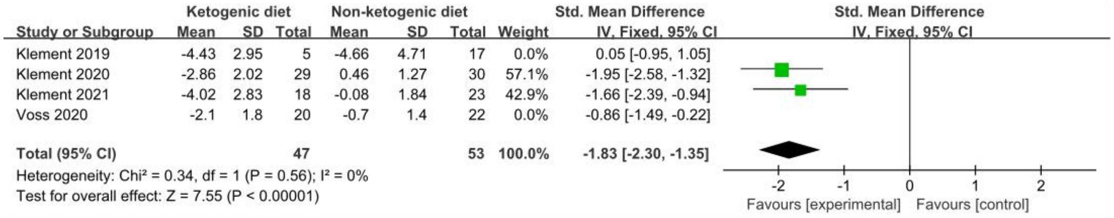

(C) BMI: post-intervention versus baseline

Study or Subgroup	Ketogenic diet Mean	SD	Total	Non-ketogenic diet Mean	SD	Total	Weight	Std. Mean Difference IV, Random, 95% CI
**1.2.1 Baseline**								
Augustus 2020	22.22	2.33	16	22.82	2.3	20	25.6%	-0.25 [-0.91, 0.41]
Kämmerer 2021	24.21	4.27	20	27.21	5.75	25	30.9%	-0.57 [-1.17, 0.03]
Khodabakhshi 2019	27.8	5.7	30	28.44	5.8	30	43.5%	-0.11 [-0.62, 0.40]
Voss 2020	27	4.07	25	27.4	4.33	25	0.0%	-0.09 [-0.65, 0.46]
**Subtotal (95% CI)**			**66**			**75**	**100.0%**	**-0.29 [-0.62, 0.04]**
Heterogeneity: Tau² = 0.00; Chi² = 1.35, df = 2 (P = 0.51); I² = 0%								
Test for overall effect: Z = 1.70 (P = 0.09)								
**1.2.2 Post intervention**								
Augustus 2020	20.89	2.23	16	22.94	2.27	20	24.4%	-0.89 [-1.58, -0.20]
Kämmerer 2021	22.83	3.6	20	25.59	4.86	25	32.2%	-0.62 [-1.23, -0.02]
Khodabakhshi 2019	25.9	3.7	30	28.47	4.1	30	43.3%	-0.65 [-1.17, -0.13]
Voss 2020	27.3	3.4	20	26.9	4.3	22	0.0%	0.10 [-0.51, 0.71]
**Subtotal (95% CI)**			**66**			**75**	**100.0%**	**-0.70 [-1.04, -0.36]**
Heterogeneity: Tau² = 0.00; Chi² = 0.39, df = 2 (P = 0.82); I² = 0%								
Test for overall effect: Z = 4.01 (P < 0.0001)								

Test for subgroup differences: Chi² = 2.83, df = 1 (P = 0.09), I² = 64.6%

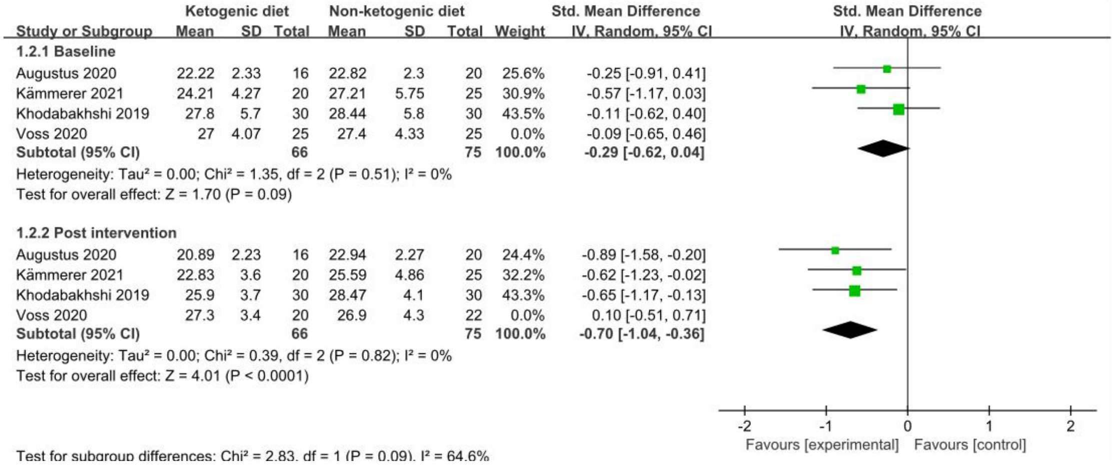

(D) Fat mass: change from baseline

Study or Subgroup	Ketogenic diet Mean	SD	Total	Non-ketogenic diet Mean	SD	Total	Weight	Std. Mean Difference IV, Random, 95% CI
Cohen 2018	-4.99	5.53	25	-2.8	5.07	20	34.3%	-0.40 [-1.00, 0.19]
Klement 2019	-1.46	1.99	4	-3.23	2.85	17	0.0%	0.62 [-0.49, 1.73]
Klement 2020	-2.07	1.59	29	0.1	1.18	30	34.5%	-1.53 [-2.12, -0.95]
Klement 2021	-3.51	2.78	17	-0.061	1.78	23	31.2%	-1.50 [-2.21, -0.78]
**Total (95% CI)**			**71**			**73**	**100.0%**	**-1.14 [-1.89, -0.38]**
Heterogeneity: Tau² = 0.34; Chi² = 8.57, df = 2 (P = 0.01); I² = 77%								
Test for overall effect: Z = 2.96 (P = 0.003)								

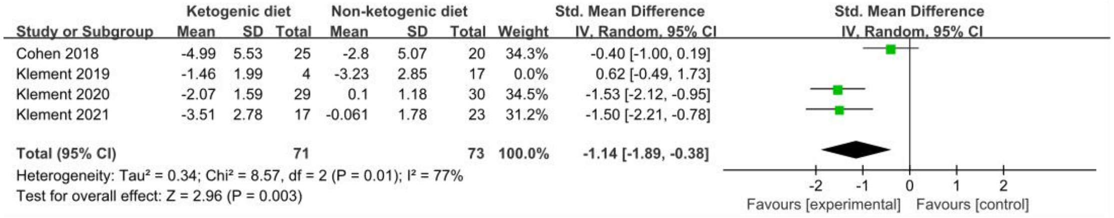

(E) Fat mass: change from baseline

Study or Subgroup	Ketogenic diet Mean	SD	Total	Non-ketogenic diet Mean	SD	Total	Weight	Std. Mean Difference IV, Random, 95% CI
Cohen 2018	-4.99	5.53	25	-2.8	5.07	20	0.0%	-0.40 [-1.00, 0.19]
Klement 2019	-1.46	1.99	4	-3.23	2.85	17	0.0%	0.62 [-0.49, 1.73]
Klement 2020	-2.07	1.59	29	0.1	1.18	30	59.9%	-1.53 [-2.12, -0.95]
Klement 2021	-3.51	2.78	17	-0.061	1.78	23	40.1%	-1.50 [-2.21, -0.78]
**Total (95% CI)**			**46**			**53**	**100.0%**	**-1.52 [-1.97, -1.07]**

Heterogeneity: Tau² = 0.00; Chi² = 0.01, df = 1 (P = 0.94); I² = 0%

Test for overall effect: Z = 6.57 (P < 0.00001)

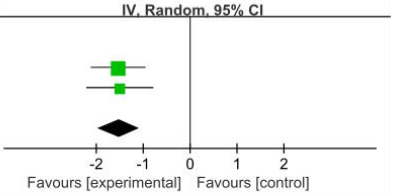

Figure S2 Supplementary analysis of effects on blood glucose, insulin and IGF-1

(A) Blood glucose: post-intervention versus baseline

Study or Subgroup	Ketogenic diet Mean	SD	Total	Non-ketogenic diet Mean	SD	Total	Weight	Std. Mean Difference IV, Random, 95% CI
**1.4.1 Baseline**								
Augustus 2020	86.15	8.56	16	85	5.9	20	0.0%	0.16 [-0.50, 0.81]
Cohen 2018	102.3	17.9	23	102.2	16.1	20	21.3%	0.01 [-0.59, 0.60]
Kämmerer 2021	86.53	1.46	20	87.28	1.35	25	21.3%	-0.53 [-1.13, 0.07]
Kang 2019	99.11	83.04	9	122.32	88.4	9	11.8%	-0.26 [-1.19, 0.67]
Khodabakhshi 2019	110.4	11.8	30	106.4	27.2	30	0.0%	0.19 [-0.32, 0.70]
Klement 2020	104.76	23.2	29	96.24	7.84	30	24.9%	0.49 [-0.03, 1.01]
Klement 2021	115.44	39.55	18	118.07	51.59	23	20.6%	-0.06 [-0.67, 0.56]
**Subtotal (95% CI)**			**99**			**107**	**100.0%**	**-0.03 [-0.40, 0.33]**
Heterogeneity: Tau² = 0.07; Chi² = 6.71, df = 4 (P = 0.15); I² = 40%								
Test for overall effect: Z = 0.17 (P = 0.87)								
**1.4.2 Post intervention**								
Augustus 2020	74.8	6.204	16	84.4	7.358	20	0.0%	-1.37 [-2.10, -0.63]
Cohen 2018	93	15.1	23	98.7	11.2	20	21.0%	-0.42 [-1.02, 0.19]
Kämmerer 2021	87.54	1.58	20	87.87	1.57	25	21.7%	-0.21 [-0.80, 0.38]
Kang 2019	132.14	246.43	9	106.25	77.68	9	12.4%	0.13 [-0.79, 1.06]
Khodabakhshi 2019	84.5	11.3	30	105.2	15.8	30	0.0%	-1.49 [-2.06, -0.91]
Klement 2020	103.15	16.29	29	96.76	9.31	30	24.5%	0.48 [-0.04, 1.00]
Klement 2021	108.73	22.51	18	131.27	61.96	23	20.4%	-0.45 [-1.08, 0.17]
**Subtotal (95% CI)**			**99**			**107**	**100.0%**	**-0.09 [-0.47, 0.29]**
Heterogeneity: Tau² = 0.09; Chi² = 7.37, df = 4 (P = 0.12); I² = 46%								
Test for overall effect: Z = 0.46 (P = 0.64)								

Test for subgroup differences: Chi² = 0.05, df = 1 (P = 0.83), I² = 0%

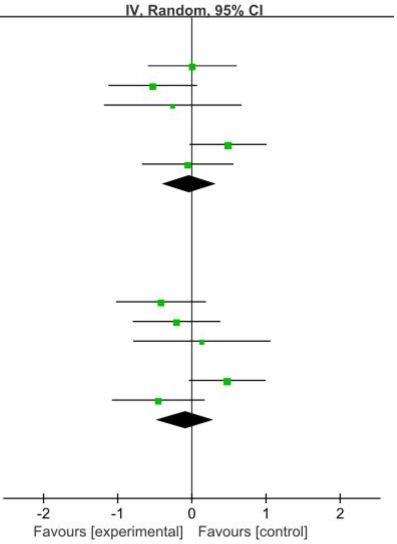

(B) Insulin: post-intervention versus baseline

Study or Subgroup	Ketogenic diet Mean	SD	Total	Non-ketogenic diet Mean	SD	Total	Weight	Std. Mean Difference IV, Random, 95% CI
**1.5.1 Baseline**								
Cohen 2018	10.5	6.4	23	14.2	9.8	20	16.1%	-0.45 [-1.05, 0.16]
Kämmerer 2021	12.79	1.89	20	16.61	2.35	24	0.0%	-1.74 [-2.45, -1.04]
Kang 2019	26.67	52.63	9	33.16	31.05	9	6.9%	-0.14 [-1.07, 0.78]
Khodabakhshi 2019	9	6.6	30	8.2	7.3	30	23.2%	0.11 [-0.39, 0.62]
Klement 2020	11.6	10.76	29	10.16	6.05	30	22.8%	0.16 [-0.35, 0.67]
Klement 2021	11.43	7.25	18	17.71	14.8	23	15.1%	-0.51 [-1.14, 0.12]
Voss 2020	12.6	5.2	20	13.22	7.61	21	15.9%	-0.09 [-0.71, 0.52]
**Subtotal (95% CI)**			**129**			**133**	**100.0%**	**-0.11 [-0.35, 0.13]**
Heterogeneity: Tau² = 0.00; Chi² = 4.58, df = 5 (P = 0.47); I² = 0%								
Test for overall effect: Z = 0.88 (P = 0.38)								
**1.5.2 Post intervention**								
Cohen 2018	6.7	4.1	23	12.1	6.7	20	16.9%	-0.97 [-1.61, -0.33]
Kämmerer 2021	12.11	1.6	20	16.28	1.89	24	0.0%	-2.32 [-3.10, -1.54]
Kang 2019	11.58	10.52	9	6.14	2.63	9	12.5%	0.68 [-0.28, 1.63]
Khodabakhshi 2019	5.7	4	30	6.9	4.5	30	18.8%	-0.28 [-0.79, 0.23]
Klement 2020	9.29	7.6	29	8.35	4.97	30	18.8%	0.14 [-0.37, 0.66]
Klement 2021	8.89	5.52	18	20.45	12.5	23	16.4%	-1.13 [-1.79, -0.46]
Voss 2020	6.9	3.7	22	17.25	19.17	18	16.7%	-0.77 [-1.42, -0.13]
**Subtotal (95% CI)**			**131**			**130**	**100.0%**	**-0.42 [-0.91, 0.07]**
Heterogeneity: Tau² = 0.27; Chi² = 18.33, df = 5 (P = 0.003); I² = 73%								
Test for overall effect: Z = 1.66 (P = 0.10)								

Test for subgroup differences: Chi² = 1.21, df = 1 (P = 0.27), I² = 17.4%

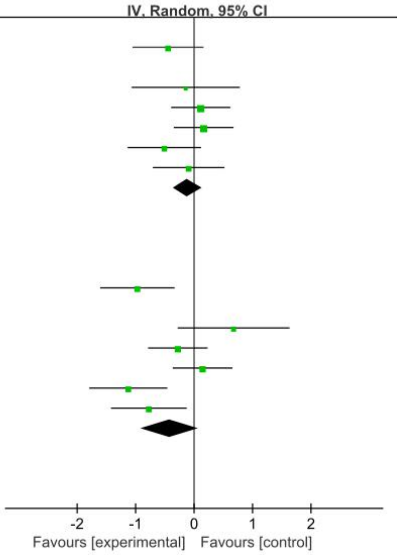

(C) Insulin: post-intervention versus baseline

Study or Subgroup	Ketogenic diet Mean	SD	Total	Non-ketogenic diet Mean	SD	Total	Weight	Std. Mean Difference IV, Random, 95% CI
**1.5.1 Baseline**								
Cohen 2018	10.5	6.4	23	14.2	9.8	20	29.9%	-0.45 [-1.05, 0.16]
Kämmerer 2021	12.79	1.89	20	16.61	2.35	24	0.0%	-1.74 [-2.45, -1.04]
Kang 2019	26.67	52.63	9	33.16	31.05	9	12.9%	-0.14 [-1.07, 0.78]
Khodabakhshi 2019	9	6.6	30	8.2	7.3	30	0.0%	0.11 [-0.39, 0.62]
Klement 2020	11.6	10.76	29	10.16	6.05	30	0.0%	0.16 [-0.35, 0.67]
Klement 2021	11.43	7.25	18	17.71	14.8	23	28.0%	-0.51 [-1.14, 0.12]
Voss 2020	12.6	5.2	20	13.22	7.61	21	29.3%	-0.09 [-0.71, 0.52]
**Subtotal (95% CI)**			**70**			**73**	**100.0%**	**-0.32 [-0.65, 0.01]**
Heterogeneity: Tau² = 0.00; Chi² = 1.18, df = 3 (P = 0.76); I² = 0%								
Test for overall effect: Z = 1.90 (P = 0.06)								
**1.5.2 Post intervention**								
Cohen 2018	6.7	4.1	23	12.1	6.7	20	23.7%	-0.97 [-1.61, -0.33]
Kämmerer 2021	12.11	1.6	20	16.28	1.89	24	0.0%	-2.32 [-3.10, -1.54]
Kang 2019	11.58	10.52	9	6.14	2.63	9	0.0%	0.68 [-0.28, 1.63]
Khodabakhshi 2019	5.7	4	30	6.9	4.5	30	30.7%	-0.28 [-0.79, 0.23]
Klement 2020	9.29	7.6	29	8.35	4.97	30	0.0%	0.14 [-0.37, 0.66]
Klement 2021	8.89	5.52	18	20.45	12.5	23	22.3%	-1.13 [-1.79, -0.46]
Voss 2020	6.9	3.7	22	17.25	19.17	18	23.2%	-0.77 [-1.42, -0.13]
**Subtotal (95% CI)**			**93**			**91**	**100.0%**	**-0.75 [-1.14, -0.36]**
Heterogeneity: Tau² = 0.06; Chi² = 4.94, df = 3 (P = 0.18); I² = 39%								
Test for overall effect: Z = 3.74 (P = 0.0002)								

Test for subgroup differences: Chi² = 2.65, df = 1 (P = 0.10), I² = 62.2%

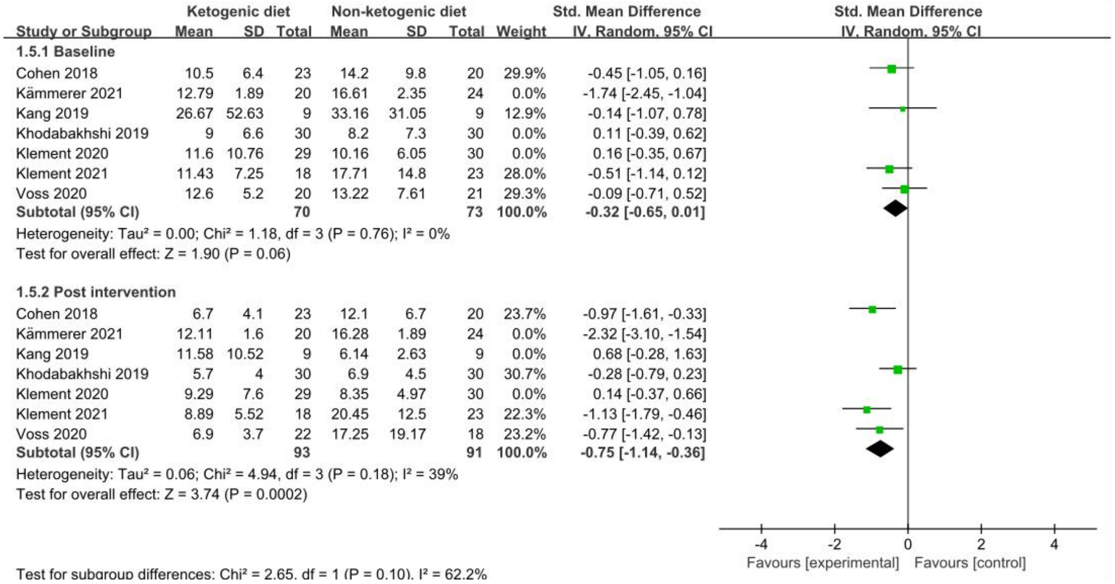

(D) IGF-1: post-intervention versus baseline

Study or Subgroup	Ketogenic diet Mean	SD	Total	Non-ketogenic diet Mean	SD	Total	Weight	Std. Mean Difference IV, Random, 95% CI
**1.6.1 Baseline**								
Cohen 2018	120.3	40.8	23	116	61.3	20	17.8%	0.08 [-0.52, 0.68]
Kämmerer 2021	14.44	12.28	20	7.37	5.27	25	0.0%	0.77 [0.16, 1.38]
Khodabakhshi 2019	151	52	30	147	56	30	25.0%	0.07 [-0.43, 0.58]
Klement 2020	198.82	78.72	29	216.76	56.85	30	24.4%	-0.26 [-0.77, 0.25]
Klement 2021	182.75	65.05	18	173.72	65.33	23	16.8%	0.14 [-0.48, 0.75]
Voss 2020	207.1	78.3	20	183.2	85.34	19	16.1%	0.29 [-0.35, 0.92]
**Subtotal (95% CI)**			**120**			**122**	**100.0%**	**0.04 [-0.21, 0.29]**
Heterogeneity: Tau² = 0.00; Chi² = 2.01, df = 4 (P = 0.73); I² = 0%								
Test for overall effect: Z = 0.30 (P = 0.76)								
**1.6.2 Post intervention**								
Cohen 2018	100.7	44	23	111	59.9	20	18.2%	-0.19 [-0.80, 0.41]
Kämmerer 2021	13.86	12.12	20	7.75	4.99	25	0.0%	0.68 [0.07, 1.28]
Khodabakhshi 2019	133	61	30	150	48	30	25.3%	-0.31 [-0.81, 0.20]
Klement 2020	187.24	92.53	29	186.46	63.72	30	25.2%	0.01 [-0.50, 0.52]
Klement 2021	135.77	47.21	18	175.72	79.33	23	16.5%	-0.58 [-1.21, 0.05]
Voss 2020	179.9	79.7	22	219.6	104.3	15	14.9%	-0.43 [-1.09, 0.23]
**Subtotal (95% CI)**			**122**			**118**	**100.0%**	**-0.27 [-0.53, -0.01]**
Heterogeneity: Tau² = 0.00; Chi² = 2.40, df = 4 (P = 0.66); I² = 0%								
Test for overall effect: Z = 2.07 (P = 0.04)								

Test for subgroup differences: Chi² = 2.83, df = 1 (P = 0.09), I² = 64.6%

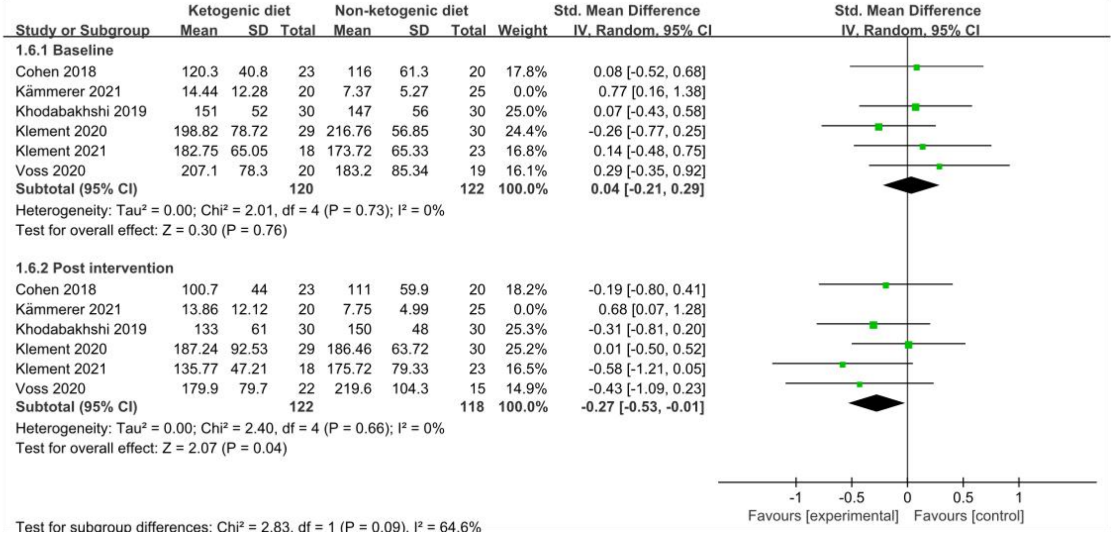

(E) IGF-1: change from baseline

Study or Subgroup	Ketogenic diet Mean	SD	Total	Non-ketogenic diet Mean	SD	Total	Weight	Std. Mean Difference IV, Random, 95% CI
Klement 2020	-22.96	57.85	29	-13.48	39.34	30	61.7%	-0.19 [-0.70, 0.32]
Klement 2021	-18.18	47.39	17	5.1	47.78	21	38.3%	-0.48 [-1.13, 0.17]
Voss 2020	24.9	50.1	20	-5.2	53.2	22		Not estimable
**Total (95% CI)**			**46**			**51**	**100.0%**	**-0.30 [-0.70, 0.10]**
Heterogeneity: Tau² = 0.00; Chi² = 0.47, df = 1 (P = 0.49); I² = 0%								
Test for overall effect: Z = 1.46 (P = 0.14)								

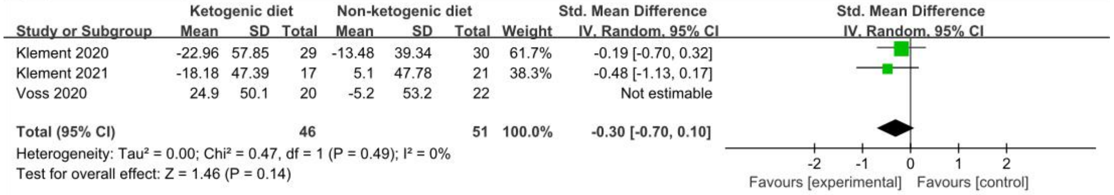

Figure S3 Supplementary analysis of effects on lipid profiles

(A) Total cholesterol: post-intervention versus baseline

Study or Subgroup	Ketogenic diet Mean	SD	Total	Non-ketogenic diet Mean	SD	Total	Weight	Std. Mean Difference IV, Random, 95% CI
**1.7.1 Baseline**								
Augustus 2020	145.1	31.43	16	159.5	29.39	20	0.0%	-0.46 [-1.13, 0.20]
Cohen 2018	205	37	23	211	43	20	23.7%	-0.15 [-0.75, 0.45]
Kämmerer 2021	233.98	8.54	20	217.5	5.79	25	0.0%	2.27 [1.50, 3.03]
Kang 2019	184.7	46.1	9	155.8	46.2	9	12.8%	0.60 [-0.35, 1.55]
Klement 2020	224.18	32.08	29	199.78	37.49	30	27.2%	0.69 [0.16, 1.22]
Klement 2021	206.91	37.33	18	210.65	36.81	23	22.9%	-0.10 [-0.72, 0.52]
OK 2018	180.2	45.7	10	155.7	46.1	9	13.4%	0.51 [-0.41, 1.43]
**Subtotal (95% CI)**			**89**			**91**	**100.0%**	**0.27 [-0.11, 0.66]**
Heterogeneity: Tau² = 0.07; Chi² = 6.38, df = 4 (P = 0.17); I² = 37%								
Test for overall effect: Z = 1.39 (P = 0.17)								
**1.7.2 Post intervention**								
Augustus 2020	132.35	30.94	16	155.3	28.07	20	0.0%	-0.76 [-1.45, -0.08]
Cohen 2018	214	60	23	208	44	20	24.4%	0.11 [-0.49, 0.71]
Kämmerer 2021	248.72	11.76	20	211.1	4.72	25	0.0%	4.31 [3.21, 5.41]
Kang 2019	180	27.1	9	156	48.5	9	9.7%	0.58 [-0.37, 1.53]
Klement 2020	220.27	47.62	29	196.92	33.08	30	32.3%	0.56 [0.04, 1.08]
Klement 2021	184.26	28.54	18	182.89	40.7	23	23.1%	0.04 [-0.58, 0.65]
OK 2018	173.8	32.1	10	156	48.4	9	10.5%	0.42 [-0.49, 1.33]
**Subtotal (95% CI)**			**89**			**91**	**100.0%**	**0.32 [0.02, 0.61]**
Heterogeneity: Tau² = 0.00; Chi² = 2.45, df = 4 (P = 0.65); I² = 0%								
Test for overall effect: Z = 2.11 (P = 0.04)								

Test for subgroup differences: Chi² = 0.03, df = 1 (P = 0.86), I² = 0%

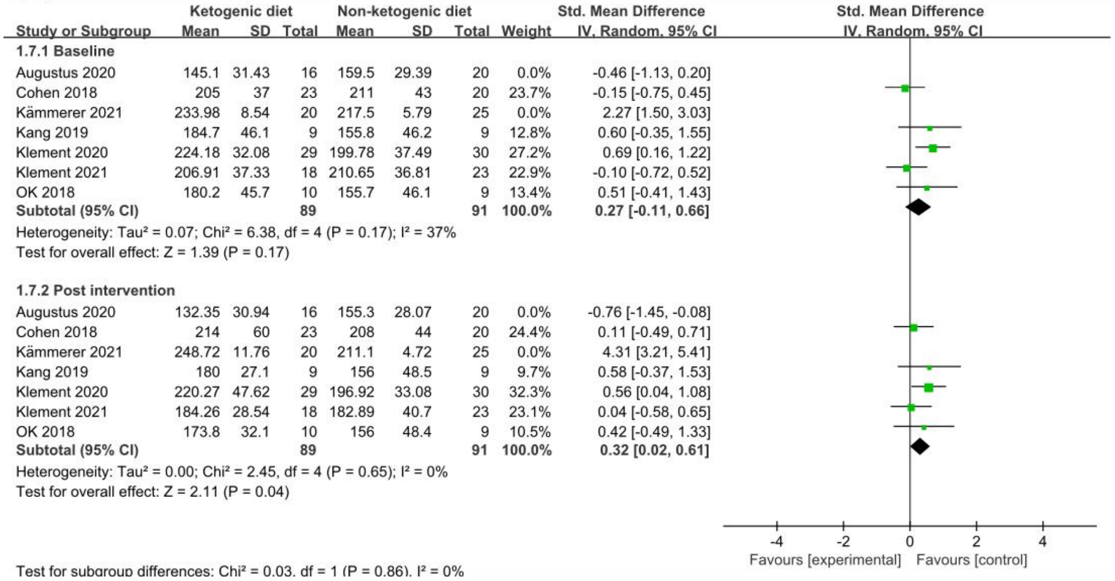

(B) HDL-C: post-intervention versus baseline

Study or Subgroup	Ketogenic diet Mean	SD	Total	Non-ketogenic diet Mean	SD	Total	Weight	Std. Mean Difference IV, Random, 95% CI
**1.8.1 Baseline**								
Cohen 2018	71	15	23	64	18	20	23.5%	0.42 [-0.19, 1.02]
Kämmerer 2021	80.18	3.8	20	65.18	2.71	25	0.0%	4.55 [3.41, 5.70]
Kang 2019	44.4	9.7	9	43.9	11.3	9	12.2%	0.05 [-0.88, 0.97]
Klement 2020	71.7	18.75	29	66.88	12.01	30	29.2%	0.30 [-0.21, 0.82]
Klement 2021	57.94	12.63	18	67.45	21.52	23	22.3%	-0.51 [-1.14, 0.11]
OK 2018	43.4	9.7	10	43.8	11.2	9	12.8%	-0.04 [-0.94, 0.86]
**Subtotal (95% CI)**			**89**			**91**	**100.0%**	**0.07 [-0.28, 0.43]**
Heterogeneity: Tau² = 0.04; Chi² = 5.41, df = 4 (P = 0.25); I² = 26%								
Test for overall effect: Z = 0.40 (P = 0.69)								
**1.8.2 Post intervention**								
Cohen 2018	72	13	23	65	15	20	23.5%	0.49 [-0.12, 1.10]
Kämmerer 2021	75.48	4.34	20	65.36	2.711	25	0.0%	2.82 [1.97, 3.67]
Kang 2019	39.6	8.53	9	42.4	6.9	9	10.6%	-0.34 [-1.28, 0.59]
Klement 2020	73.06	24.18	29	67.48	15.19	30	31.9%	0.27 [-0.24, 0.79]
Klement 2021	64.69	14.27	18	62.79	17.89	23	23.0%	0.11 [-0.50, 0.73]
OK 2018	38.4	8.8	10	42.4	6.9	9	11.0%	-0.48 [-1.40, 0.44]
**Subtotal (95% CI)**			**89**			**91**	**100.0%**	**0.14 [-0.17, 0.45]**
Heterogeneity: Tau² = 0.01; Chi² = 4.34, df = 4 (P = 0.36); I² = 8%								
Test for overall effect: Z = 0.88 (P = 0.38)								

Test for subgroup differences: Chi² = 0.08, df = 1 (P = 0.78), I² = 0%

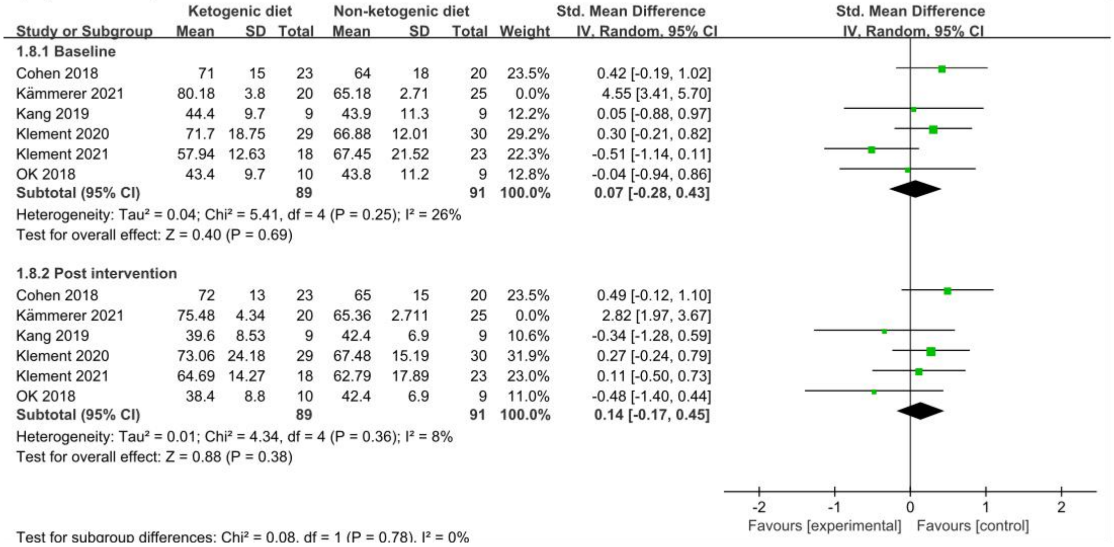

(C) HDL-C: change from baseline

Study or Subgroup	Ketogenic diet Mean	SD	Total	Non-ketogenic diet Mean	SD	Total	Weight	Std. Mean Difference IV, Random, 95% CI
Cohen 2018	1.3	13.9	23	0.8	8.9	20	50.5%	0.04 [-0.56, 0.64]
Klement 2021	5.38	10.83	18	-2.87	6.96	20		Not estimable
Voss 2020	-2	7.7	20	-1.9	9	22	49.5%	-0.01 [-0.62, 0.59]
**Total (95% CI)**			**43**			**42**	**100.0%**	**0.02 [-0.41, 0.44]**
Heterogeneity: Tau² = 0.00; Chi² = 0.01, df = 1 (P = 0.90); I² = 0%								
Test for overall effect: Z = 0.07 (P = 0.94)								

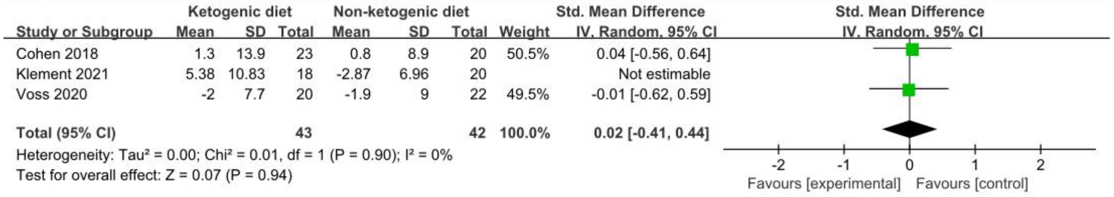

(D) LDL-C: post-intervention versus baseline

Study or Subgroup	Ketogenic diet Mean	SD	Total	Non-ketogenic diet Mean	SD	Total	Weight	Std. Mean Difference IV, Random, 95% CI
**1.9.1 Baseline**								
Cohen 2018	112	31	23	119	34	20	23.9%	-0.21 [-0.81, 0.39]
Kämmerer 2021	141.97	6.93	20	137.23	5.48	25		Not estimable
Kang 2019	112.9	38.6	9	86	36.5	9	12.6%	0.68 [-0.28, 1.64]
Klement 2020	143.36	39.48	29	117.4	29.19	30	27.5%	0.74 [0.21, 1.27]
Klement 2021	133.45	31.29	18	116.28	36.55	23	22.7%	0.49 [-0.14, 1.12]
OK 2018	110.8	37	10	86	36.5	9	13.2%	0.64 [-0.29, 1.57]
**Subtotal (95% CI)**			**89**			**91**	**100.0%**	**0.44 [0.05, 0.82]**
Heterogeneity: Tau² = 0.07; Chi² = 6.20, df = 4 (P = 0.18); I² = 36%								
Test for overall effect: Z = 2.22 (P = 0.03)								
**1.9.2 Post intervention**								
Cohen 2018	123	55	23	119	40	20	25.2%	0.08 [-0.52, 0.68]
Kämmerer 2021	158.39	8.39	20	128.83	5.11	25		Not estimable
Kang 2019	112	27.6	9	97.4	41.9	9	10.4%	0.39 [-0.54, 1.33]
Klement 2020	141.26	47.38	29	113.82	26.83	25	29.8%	0.69 [0.14, 1.24]
Klement 2021	109.47	26.62	18	103.03	35	23	23.7%	0.20 [-0.42, 0.82]
OK 2018	108.4	28.3	10	97.4	41.9	9	11.0%	0.30 [-0.61, 1.20]
**Subtotal (95% CI)**			**89**			**86**	**100.0%**	**0.35 [0.05, 0.65]**
Heterogeneity: Tau² = 0.00; Chi² = 2.47, df = 4 (P = 0.65); I² = 0%								
Test for overall effect: Z = 2.25 (P = 0.02)								

Test for subgroup differences: Chi² = 0.13, df = 1 (P = 0.72), I² = 0%

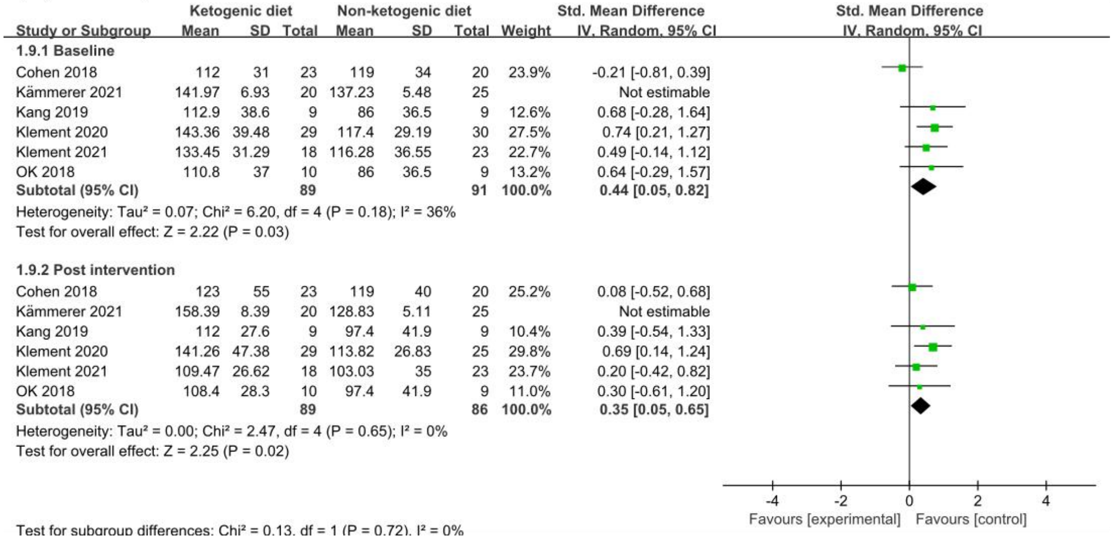

(E) Triglyceride: post-intervention versus baseline

Study or Subgroup	Ketogenic diet Mean	SD	Total	Non-ketogenic diet Mean	SD	Total	Weight	Std. Mean Difference IV, Random, 95% CI
**1.10.1 Baseline**								
Cohen 2018	108	48	23	134	59	20	29.5%	-0.48 [-1.09, 0.13]
Kämmerer 2021	78.24	5.11	20	108.95	8.59	25	0.0%	-4.16 [-5.23, -3.08]
Kang 2019	137.9	89	9	120.8	74.5	9	17.8%	0.20 [-0.73, 1.13]
Klement 2020	115.4	41.21	29	96.77	40.93	30	34.3%	0.45 [-0.07, 0.96]
Klement 2021	107.31	18.5	18	120.03	51.07	23	0.0%	-0.31 [-0.93, 0.31]
OK 2018	131.8	86.1	10	120.7	74.5	9	18.4%	0.13 [-0.77, 1.03]
**Subtotal (95% CI)**			**71**			**68**	**100.0%**	**0.07 [-0.39, 0.54]**
Heterogeneity: Tau² = 0.10; Chi² = 5.25, df = 3 (P = 0.15); I² = 43%								
Test for overall effect: Z = 0.30 (P = 0.76)								
**1.10.2 Post intervention**								
Cohen 2018	97	33	23	111	46	20	29.8%	-0.35 [-0.95, 0.26]
Kämmerer 2021	76.78	5.38	20	109.25	11.31	25	0.0%	-3.48 [-4.43, -2.52]
Kang 2019	131	49.7	9	110.6	51.2	9	17.8%	0.39 [-0.55, 1.32]
Klement 2020	80.24	24.43	29	108.53	55.63	30	33.8%	-0.65 [-1.17, -0.12]
Klement 2021	81.32	58.19	18	136.9	36.55	23	0.0%	-1.15 [-1.82, -0.48]
OK 2018	125.7	49.8	10	110.5	51.1	9	18.6%	0.29 [-0.62, 1.19]
**Subtotal (95% CI)**			**71**			**68**	**100.0%**	**-0.20 [-0.67, 0.28]**
Heterogeneity: Tau² = 0.10; Chi² = 5.37, df = 3 (P = 0.15); I² = 44%								
Test for overall effect: Z = 0.82 (P = 0.41)								

Test for subgroup differences: Chi² = 0.64, df = 1 (P = 0.42), I² = 0%

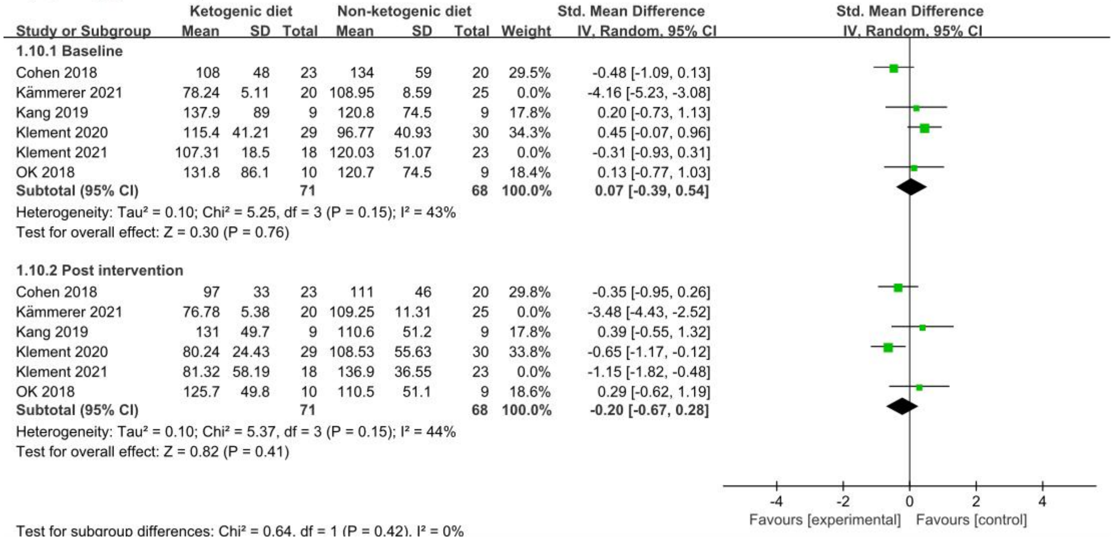

(F) Triglyceride: change from baseline

Study or Subgroup	Ketogenic diet Mean	SD	Total	Non-ketogenic diet Mean	SD	Total	Weight	Std. Mean Difference IV, Random, 95% CI
Cohen 2018	-11.3	40.7	23	-22.8	35.1	20	0.0%	0.30 [-0.31, 0.90]
Klement 2020	-21.92	42.72	29	-0.08	35.69	30	39.6%	-0.55 [-1.07, -0.03]
Klement 2021	6.11	30.6	18	15.84	45.49	22	30.9%	-0.24 [-0.87, 0.38]
Voss 2020	-33.7	45.6	20	9.7	38.4	22	29.4%	-1.01 [-1.66, -0.37]
**Total (95% CI)**			**67**			**74**	**100.0%**	**-0.59 [-1.00, -0.18]**
Heterogeneity: Tau² = 0.04; Chi² = 2.87, df = 2 (P = 0.24); I² = 30%								
Test for overall effect: Z = 2.82 (P = 0.005)								

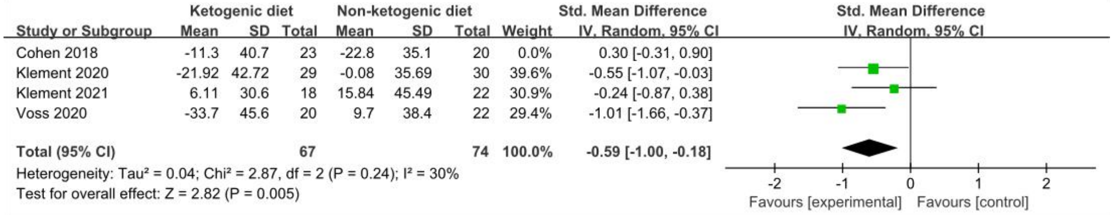

Figure S4 Supplementary analysis of effects on ketosis

(A) Ketone body: post-intervention versus baseline

Study or Subgroup	Ketogenic diet Mean	SD	Total	Non-ketogenic diet Mean	SD	Total	Weight	Std. Mean Difference IV, Random, 95% CI
**1.11.1 Baseline**								
Kang 2019	2.13	0.793	9	2.07	1.14	9	0.0%	0.06 [-0.87, 0.98]
Khodabakhshi 2019	0.007	0.025	30	0.003	0.018	30	100.0%	0.18 [-0.33, 0.69]
**Subtotal (95% CI)**			**30**			**30**	**100.0%**	**0.18 [-0.33, 0.69]**
Heterogeneity: Not applicable								
Test for overall effect: Z = 0.70 (P = 0.48)								
**1.11.2 Post intervention**								
Kang 2019	1.67	0.952	9	2.3	1.59	9	0.0%	-0.46 [-1.40, 0.48]
Khodabakhshi 2019	0.923	0.699	30	0.007	0.026	30	100.0%	1.83 [1.22, 2.44]
**Subtotal (95% CI)**			**30**			**30**	**100.0%**	**1.83 [1.22, 2.44]**
Heterogeneity: Not applicable								
Test for overall effect: Z = 5.89 (P < 0.00001)								

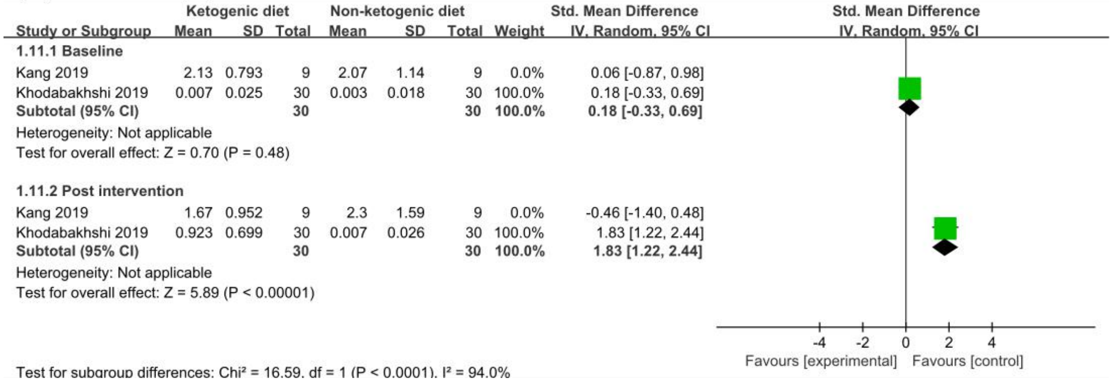

Test for subgroup differences: Chi² = 16.59, df = 1 (P < 0.0001), I² = 94.0%

(B) β-hydroxybutyrate: post-intervention versus baseline

Study or Subgroup	Ketogenic diet Mean	SD	Total	Non-ketogenic diet Mean	SD	Total	Weight	Std. Mean Difference IV, Random, 95% CI
**1.12.1 Baseline**								
Cohen 2018	0.36	0.69	23	0.2	0.09	20	32.2%	0.31 [-0.29, 0.91]
Kang 2019	0.142	0.057	9	0.116	0.064	9	13.4%	0.41 [-0.53, 1.34]
Klement 2019	0.3015	0.3645	5	0.2029	0.1225	17	11.5%	0.48 [-0.53, 1.49]
Klement 2020	0.139	0.109	29	0.083	0.066	30	42.9%	0.62 [0.09, 1.14]
Klement 2021	0.26	0.222	18	0.167	0.148	23	0.0%	0.50 [-0.13, 1.12]
**Subtotal (95% CI)**			**66**			**76**	**100.0%**	**0.47 [0.13, 0.82]**
Heterogeneity: Tau² = 0.00; Chi² = 0.59, df = 3 (P = 0.90); I² = 0%								
Test for overall effect: Z = 2.71 (P = 0.007)								
**1.12.2 Post intervention**								
Cohen 2018	0.91	0.73	23	0.25	0.18	20	29.6%	1.18 [0.53, 1.83]
Kang 2019	0.286	0.165	9	0.273	0.152	9	18.0%	0.08 [-0.85, 1.00]
Klement 2019	1.6176	1.9778	4	0.7988	1.1608	17	13.4%	0.59 [-0.51, 1.70]
Klement 2020	0.67	0.553	29	0.357	0.632	30	38.9%	0.52 [-0.00, 1.04]
Klement 2021	0.862	0.609	18	0.137	0.119	23	0.0%	1.73 [0.99, 2.46]
**Subtotal (95% CI)**			**65**			**76**	**100.0%**	**0.65 [0.20, 1.09]**
Heterogeneity: Tau² = 0.06; Chi² = 4.25, df = 3 (P = 0.24); I² = 29%								
Test for overall effect: Z = 2.86 (P = 0.004)								

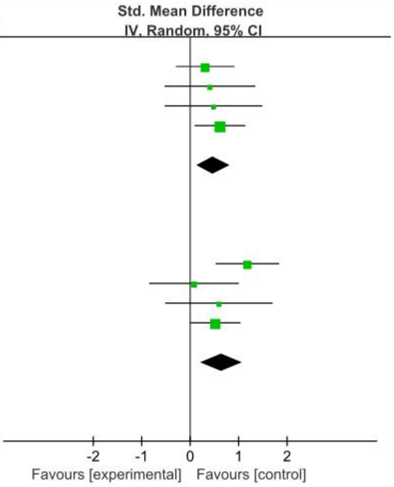

Test for subgroup differences: Chi² = 0.36, df = 1 (P = 0.55), I² = 0%

Figure S5 Supplementary analysis of effects on creatinine

(A) Creatinine: post-intervention versus baseline

Study or Subgroup	Ketogenic diet Mean	SD	Total	Non-ketogenic diet Mean	SD	Total	Weight	Std. Mean Difference IV, Random, 95% CI
**1.13.1 Baseline**								
Kämmerer 2021	0.342	0.025	20	0.338	0.035	25	27.9%	0.13 [-0.46, 0.72]
Kang 2019	131.8	87.7	9	65.4	54.5	9	13.2%	0.87 [-0.11, 1.84]
Khodabakhshi 2019	1.4	0.001	30	0.88	0.15	30	0.0%	4.84 [3.81, 5.87]
Klement 2020	0.808	713	30	0.842	0.133	29	33.0%	-0.00 [-0.51, 0.51]
Klement 2021	0.873	0.19	18	0.944	0.189	23	25.9%	-0.37 [-0.99, 0.25]
**Subtotal (95% CI)**			**77**			**86**	**100.0%**	**0.05 [-0.34, 0.45]**
Heterogeneity: Tau² = 0.05; Chi² = 4.49, df = 3 (P = 0.21); I² = 33%								
Test for overall effect: Z = 0.27 (P = 0.79)								
**1.13.2 Post intervention**								
Kämmerer 2021	0.306	0.03	20	0.36	0.032	25	25.2%	-1.70 [-2.40, -1.01]
Kang 2019	232	93.5	9	150.2	49.3	9	21.8%	1.04 [0.04, 2.04]
Khodabakhshi 2019	0.77	0.09	30	0.86	0.15	30	0.0%	-0.72 [-1.24, -0.19]
Klement 2020	0.755	0.109	30	0.839	0.123	29	26.9%	-0.71 [-1.24, -0.19]
Klement 2021	0.87	0.173	18	0.908	0.194	23	26.0%	-0.20 [-0.82, 0.42]
**Subtotal (95% CI)**			**77**			**86**	**100.0%**	**-0.45 [-1.37, 0.47]**
Heterogeneity: Tau² = 0.75; Chi² = 21.84, df = 3 (P < 0.0001); I² = 86%								
Test for overall effect: Z = 0.95 (P = 0.34)								

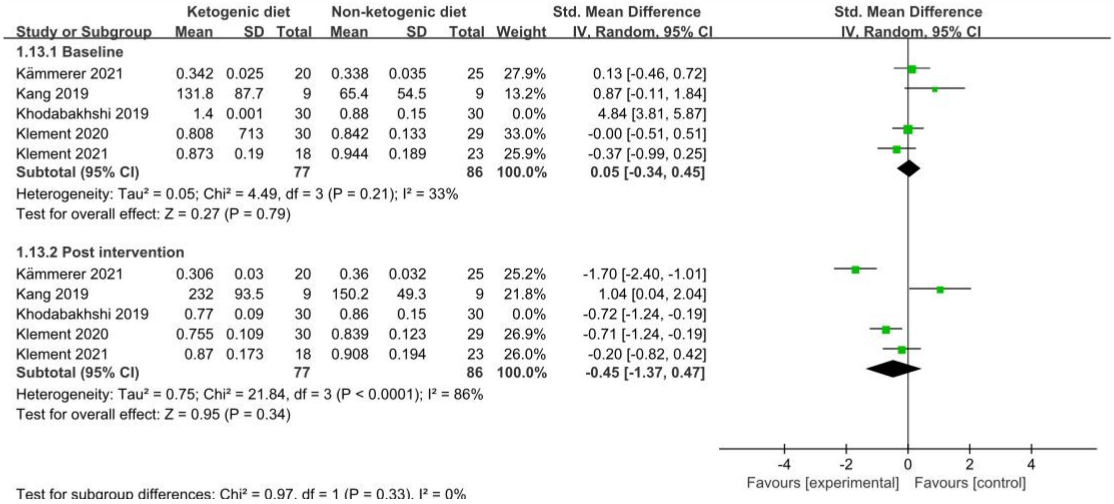

Test for subgroup differences: Chi² = 0.97, df = 1 (P = 0.33), I² = 0%

(B) Creatinine: post-intervention versus baseline

Study or Subgroup	Ketogenic diet Mean	SD	Total	Non-ketogenic diet Mean	SD	Total	Weight	Std. Mean Difference IV, Random, 95% CI
**1.13.1 Baseline**								
Kämmerer 2021	0.342	0.025	20	0.338	0.035	25	31.0%	0.13 [-0.46, 0.72]
Kang 2019	131.8	87.7	9	65.4	54.5	9	0.0%	0.87 [-0.11, 1.84]
Khodabakhshi 2019	1.4	0.001	30	0.88	0.15	30	0.0%	4.84 [3.81, 5.87]
Klement 2020	0.808	713	30	0.842	0.133	29	41.2%	-0.00 [-0.51, 0.51]
Klement 2021	0.873	0.19	18	0.944	0.189	23	27.7%	-0.37 [-0.99, 0.25]
**Subtotal (95% CI)**			**68**			**77**	**100.0%**	**-0.06 [-0.39, 0.27]**
Heterogeneity: Tau² = 0.00; Chi² = 1.38, df = 2 (P = 0.50); I² = 0%								
Test for overall effect: Z = 0.37 (P = 0.71)								
**1.13.2 Post intervention**								
Kämmerer 2021	0.306	0.03	20	0.36	0.032	25	31.6%	-1.70 [-2.40, -1.01]
Kang 2019	232	93.5	9	150.2	49.3	9	0.0%	1.04 [0.04, 2.04]
Khodabakhshi 2019	0.77	0.09	30	0.86	0.15	30	0.0%	-0.72 [-1.24, -0.19]
Klement 2020	0.755	0.109	30	0.839	0.123	29	35.2%	-0.71 [-1.24, -0.19]
Klement 2021	0.87	0.173	18	0.908	0.194	23	33.2%	-0.20 [-0.82, 0.42]
**Subtotal (95% CI)**			**68**			**77**	**100.0%**	**-0.86 [-1.65, -0.06]**
Heterogeneity: Tau² = 0.40; Chi² = 10.22, df = 2 (P = 0.006); I² = 80%								
Test for overall effect: Z = 2.11 (P = 0.04)								

Std. Mean Difference
IV, Random, 95% CI

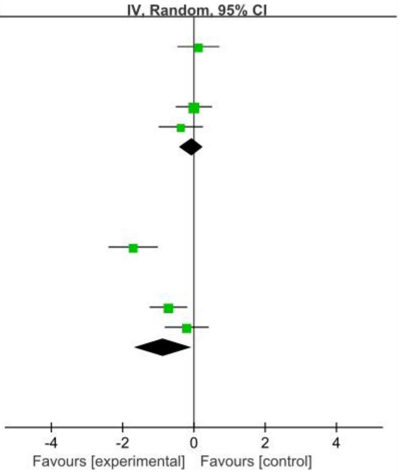

Test for subgroup differences: Chi² = 3.26, df = 1 (P = 0.07), I² = 69.3%
