# Supplementary material for: Effect of Ketogenic Diets on Body Composition and Metabolic Parameters of Cancer Patients: A Systematic Review and Meta-Analysis
Source: Nutrients. 2022 Oct 8;14(19):4192. doi: 10.3390/nu14194192 (PMC9570668; doi:10.3390/nu14194192)
Supplement: Supplementary file 1 [file nutrients-14-04192-s001.zip › Supplementaty Table S3 GRADE evidence profile.pdf]

Table S3 GRADE evidence profile-for the comparison ketogenic diets vs. non-ketogenic diets

Quality assessment								No of patients		Effect	Quality	Importance
**Item**	**No of studies**	**Design**	**Risk of bias**	**Inconsistency**	**Indirectness**	**Imprecision**	**Other considerations**	**Ketogenic diets**	**Control**	**Absolute**		
**body weght**	4	randomised trials	serious[1]	no serious inconsistency	no serious indirectness	no serious imprecision	none	72	92	SMD 1.29 lower (1.64 to 0.93 lower)	⊕⊕⊕⊝ MODERATE	CRITICAL
BMI	1	observational studies	serious[1]	no serious inconsistency	no serious indirectness	very serious[2]	none	18	23	SMD 1.3 lower (1.99 to 0.62 lower)	⊕⊝⊝⊝ VERY LOW	CRITICAL
**fat mass**	4	randomised trials	serious[1]	no serious inconsistency	no serious indirectness	no serious imprecision	none	75	90	SMD 0.78 lower (1.63 lower to 0.07 higher)	⊕⊕⊕⊝ MODERATE	CRITICAL
**total cholesterol**	2	randomised trials	serious[1]	no serious inconsistency	no serious indirectness	serious[2]	none	43	42	SMD 0.1 higher (0.33 lower to 0.52 higher)	⊕⊕⊝⊝ LOW	CRITICAL
**HDL-C**	3	randomised trials	serious[1]	serious[3]	no serious indirectness	no serious imprecision	none	61	62	SMD 0.29 higher (0.26 lower to 0.85 higher)	⊕⊕⊝⊝ LOW	CRITICAL
**LDL-C**	3	randomised trials	serious[1]	no serious inconsistency	no serious indirectness	no serious imprecision	none	61	64	SMD 0.03 higher (0.46 lower to 0.52 higher)	⊕⊕⊕⊝ MODERATE	CRITICAL
**triglycerides**	4	randomised trials	serious[1]	serious[3]	no serious indirectness	no serious imprecision	none	90	94	SMD 0.37 lower (0.89 lower to 0.15 higher)	⊕⊕⊝⊝ LOW	CRITICAL
**blood glucose**	1	randomised trials	no serious risk of bias	no serious inconsistency	no serious indirectness	very serious[2]	none	20	22	SMD 0.63 lower (1.25 to 0.01 lower)	⊕⊕⊝⊝ LOW	CRITICAL
**insulin**	3	randomised trials	serious[1]	no serious inconsistency	no serious indirectness	no serious imprecision	none	66	71	SMD 0.15 lower (0.49 lower to 0.19 higher)	⊕⊕⊕⊝ MODERATE	CRITICAL

**IGF-1**	3	randomised trials	serious[1]	no serious inconsistency	no serious indirectness	no serious imprecision	none	66	73	SMD 0.04 lower (0.38 lower to 0.3 higher)	⊕⊕⊕⊝ MODERATE	CRITICAL
**ketone body**	2	randomised trials	serious[1]	no serious inconsistency	no serious indirectness	serious[2]	none	49	52	SMD 1.25 higher (0.27 to 2.23 higher)	⊕⊕⊝⊝ LOW	CRITICAL
**β-hydroxybutyrate**	1	observational studies	serious[1]	no serious inconsistency	no serious indirectness	serious[2]	none	29	30	SMD 0.79 higher (0.26 to 1.32 higher)	⊕⊝⊝⊝ VERY LOW	IMPORTANT
**creatine**	1	observational studies	serious[1]	no serious inconsistency	no serious indirectness	serious[2]	none	29	29	SMD 0.46 lower (0.98 lower to 0.07 higher)	⊕⊝⊝⊝ VERY LOW	IMPORTANT
free T3	2	observational studies	serious[1]	no serious inconsistency	no serious indirectness	serious[2]	none	47	52	SMD 0.75 lower (1.42 to 0.09 lower)	⊕⊝⊝⊝ VERY LOW	IMPORTANT

CI: Confidence interval; SMD: Standard Mean difference

[1] There was high risk in slection bias and performance bias. Since the intervention was dietary, the intervention group was informed in all studies. And some participants were assigned to different groups according to their personal desires.

[2] Few participants.

[3] Unexplained heterogeneity.
